# Identification of Quantitative Trait Loci for Clubroot Resistance in *Brassica oleracea* With the Use of *Brassica* SNP Microarray

**DOI:** 10.3389/fpls.2018.00822

**Published:** 2018-06-18

**Authors:** Lisha Peng, Lili Zhou, Qinfei Li, Dayong Wei, Xuesong Ren, Hongyuan Song, Jiaqin Mei, Jun Si, Wei Qian

**Affiliations:** ^1^College of Horticulture and Landscape Architecture, Southwest University, Chongqing, China; ^2^Key Laboratory of Horticulture Science for Southern Mountains Regions, Ministry of Education, Chongqing, China; ^3^Chongqing Key Laboratory of Olericulture, Chongqing, China; ^4^Chongqing Yudongnan Academy of Agricultural Sciences, Chongqing, China; ^5^College of Agronomy and Biotechnology, Southwest University, Chongqing, China; ^6^Academy of Agricultural Sciences, Southwest University, Chongqing, China

**Keywords:** *Brassica oleracea*, *Plasmodiophora brassicae*, clubroot, SNP microarray, quantitative trait loci

## Abstract

Increasing clubroot resistance (CR) of *Brassica oleracea* by ascertaining the molecular mechanisms has been the key focus in modern *B. oleracea* breeding. In order to identify the quantitative trait loci (QTLs) associated with CR in *B. oleracea*, 94 F2 vegetative lines which were developed by tissue culture of selfed seeds from the F1 generation between a clubroot-resistant *B. oleracea* inbred line and a susceptible line, were identified for disease incidence and six CR-associated traits under a lab inoculation by *Plasmodiophora brassicae* and were genotyped with the 60K *Brassica* SNP array. Significant correlations were detected for numbers of fibrous roots and *P. brassicae* content in roots with disease incidence. Nine linkage groups were constructed from 565 bins which covered around 3,000 SNPs, spanning 1,028 cM of the *B. oleracea* genome with an average distance of 1.82 cM between adjacent bins. A total of 23 QTLs were identified for disease incidence and the other two correlated traits, individually explaining 6.1–17.8% of the phenotypic variation. Several overlaps were detected among traits, including one three-traits-overlapped locus on linkage group C08 and two important overlapped regions between the two CR-associated traits on C06. The QTLs were compared with known CR loci/genes and the novelty of our QTLs was discussed.

## Introduction

Clubroot, caused by the soil-borne obligate *Plasmodiophora brassicae*, is a devastating disease in *Brassica* crops including cabbage (*Brassica oleracea* L. var. *capitata*) which is one of the most important vegetable crops in the world (Hirai, 2006; Dixon, 2009). It causes serious yield loss in cabbage since the pathogen always induces galls on the plant root, thus hinders the uptake of water and nutrients and finally leads to abnormal growth (Dixon and Robinson, 2010). It is hard to control clubroot by cultural managements or chemical fungicides due to the long period of the pathogen surviving in soil. Therefore, developing resistance cultivars is the most effective way to control this disease.

Identification of clubroot resistance (CR) quantitative trait loci (QTLs) or genes is of great importance in the resistance breeding. Complete resistant accessions against specific pathogen isolates were found in European fodder turnips (*B. rapa*) and at least eight resistant loci were identified in *B. rapa* including *Crr1*, *Crr2*, *Crr3*, *Crr4*, *CRa*, *CRb*, *CRc*, and *CRk* (Matsumoto et al., 1998; Piao et al., 2002, 2004; Hirai et al., 2004; Saito et al., 2006; Suwabe et al., 2006; Sakamoto et al., 2008; Hatakeyama et al., 2013; Kato et al., 2013; Huang et al., 2017; Yu et al., 2017). These loci were reported to control CR in a qualitative plus quantitative manner (Piao et al., 2002; Suwabe et al., 2006; Sakamoto et al., 2008). Likewise, a few CR QTLs, such as *CR2a*, *CR2b*, *pb-3*, *pb-4*, *pb-Bol* and *PbBo(Anju)1*∼*4*, were identified from *B. oleracea* (Landry et al., 1992; Voorrips et al., 1997; Rocherieux et al., 2004; Moriguchi et al., 2010; Nagaoka et al., 2010; Lee et al., 2016) which might be partially resistant to clubroot (Crisp et al., 1989; Piao et al., 2009). Being differently, the CR trait is possibly controlled by many quantitative loci in *B. oleracea* (Piao et al., 2009; Nagaoka et al., 2010; Tomita et al., 2013). Therefore, identify more CR loci from different *B. oleracea* resources with wide genetic basis will be benefit to the breeding of clubroot-resistant cabbages.

In our previous identification, a cabbage inbred line was found to be highly resistant to clubroot disease, thus an F2 segregating population was developed from the cross between this line and a susceptible *B. oleracea*. On the other hand, a 60K *Brassica* SNP microarray was successfully released (Clarke et al., 2016) and used for the construction of genetic linkage map with high density of markers in *B. oleracea* (Mei et al., 2017). In the present study, disease incidence and several CR related traits were investigated in this F2 population in lab tests, and QTLs for these traits were identified by using the linkage group built by data from the SNP microarray. Our study will be helpful for clubroot resistance breeding in *B. oleracea.*

## Materials and Methods

### Plant Materials and Resistance Evaluation

An F2 segregating population, comprising of 94 cloned-lines developed by tissue culture, was developed from hybridization between two inbred lines of *B. oleracea*, ‘263’ and ‘GZ87’ with diverse resistance levels against *P. brassicae*. The development of clones for each F2 genotype was conducted according to Luo et al. (2000). The vegetative plants were transplanted into 90 mm × 80 mm pots after rooting and kept in a climate chamber for 1 week (16/8 h light/dark cycle under 26/20°C). Then each plant was inoculated by watering 2 mL *P. brassicae* (the 4^th^ race) resting spores suspension (2 × 10^8^ spores/mL) at the stem base using a pipette method (Carlsson et al., 2004; Nagaoka et al., 2010) and kept in the climate chamber with the same condition as before. Phenotypic data were collected at 6 weeks post-inoculation, including disease incidence (DIC, percentage of diseased plants in total plants), fresh weight per plant (FW), fibrous root weight/root weight (FR/R), length of root (LR), number of fibrous roots (NFRs), ratio of root surface covered with fibrous roots (RFR), and *P. brassicae* content in roots (PCR). Four biological replicates of resistance test were conducted in two rounds. Ten to twenty two plants per line were tested in each replicate for the calculation of DIC and five plants for other six indexes. Pearson’s simple correlations were calculated between traits of interest via SAS software (SAS Institute, 1999).

### Genotyping, Map Construction, and QTL Analysis

Genomic DNA was extracted from young leaf of plant using the CTAB method (Allen et al., 2006). DNA samples of two parental lines and the 94 F2 lines were genotyped by the *Brassica* 60K Bead Chip Array (Infinium^®^, Illumina, Inc., San Diego, CA, United States) (Clarke et al., 2016). SNPs were aligned to the reference genomes of *B. oleracea* (version 1.1)^1^ using a local BLAST search. Bins were developed for each chromosome using Perl language by combining SNPs with identical genotypes across the F2 population (Zhang et al., 2014) and then used for genetic map construction in IciMapping version 4.1 with default parameters (Wang et al., 2016). QTLs were analyzed using the inclusive composite interval mapping (ICIM) model in IciMapping with optimized parameters (Step = 1 cM; PIN = 0.005). A permutation test with 1,000 permutations was performed for each trait to calculate the threshold of LOD score at the significance level of *P* = 0.05.

## Results

### Phenotypic Performance of Parents and F2 Lines

**FIGURE 1:**
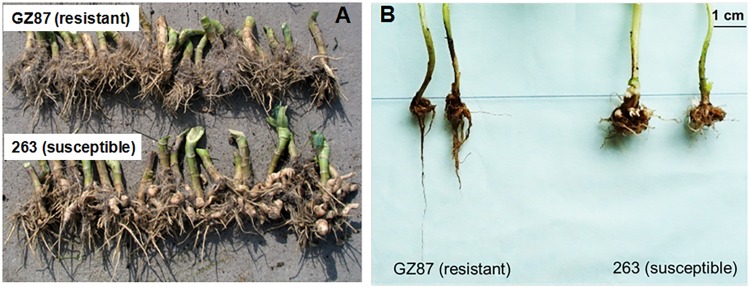
The root morphology of ‘GZ87’ and ‘263’ after infection by *Plasmodiophora brassicae* under field **(A)** and lab **(B)** assays.

According to the field identification for three successive years at Fuling, Chongqing, China (E107.6459, N29.5709) where happens serious clubroot disease every year caused mainly by the 4^th^ race of *P. brassicae* (identified according to Williams host system, data not shown), ‘GZ87’ exhibited complete resistance with a disease index of 0, while ‘263’ which is a founder parent in our cabbage breeding program showed stable susceptibility to *P. brassicae* (with average DIC of 72.6% and disease index of 33.7). Under the lab identification, similarly, ‘GZ87’ exhibited obviously higher resistance (DIC = 0) than ‘263’ (DIC = 80.8) (**Figure 1**). Wide variations were detected among the F2 lines for all the seven traits, of which FW, LR, and RFR showed normal distributions, while DIC, FR/R, NFR, and PCR exhibited skew distributions (**Table 1**). Three traits, particularly PCR and NFR, were found to be significantly correlated with DIC (*r*= -0.881 and -0.640, respectively) (**Table 2**). Therefore, QTLs were subsequently screened for DIC, PCR, and NFR.

**Table 1:** The phenotypic performance of clubroot resistance associated traits in the parental lines and the F2 population of *Brassica oleracea.*

Trait^∗^	The parental lines		The F2 population				
	GZ87	263	Maximum	Minimum	Mean (±*SD*)	Skewness	Kurtosis
DIC	0	80.8	100	0	58.40 ± 27.05	-13.11	-1.68
FW	3.24	1.37	4.47	0.19	1.63 ± 0.94	0.86	0.00
FR/R	34.07	6.15	70.33	2.01	15.95 ± 9.05	1.66	6.59
LR	6.48	2.45	11.20	0.60	3.53 ± 2.04	1.35	1.79
NFR	3.02	1.66	4.00	1.00	2.64 ± 0.95	-0.13	-1.24
RFR	3.45	1.40	3.76	1.00	1.88 ± 0.71	0.96	0.09
PCR	14.24	162.21	198.02	20.12	62.73 ± 33.52	0.93	1.20

*^∗^DIC, disease incidence; FW, fresh weight per plant; FR/R, fibrous root weight/root weight; LR, length of root; NFR, numbers of fibrous roots; RFR, ratio of root surface covered with fibrous roots; PCR, P. brassicae content in roots.*

**Table 2:** Correlation among the clubroot resistance associated traits in *Brassica oleracea.*

Trait^#^	DIC	FW	FR/R	LR	NFR	RFR
FW	-0.233^∗∗^					
FR/R	-0.006	0.239^∗∗^				
LR	-0.186	0.796^∗∗^	0.464^∗∗^			
NFR	-0.640^∗∗^	0.349^∗∗^	0.131	0.363^∗∗^		
RFR	-0.412^∗∗^	0.405^∗∗^	0.240^∗∗^	0.399^∗∗^	0.590^∗∗^	
PCR	0.881^∗∗^	-0.290^∗∗^	-0.096	-0.258^∗∗^	-0.870^∗∗^	-0.534^∗∗^

*^#^DIC, disease incidence; FW, fresh weight per plant; FR/R, fibrous root weight/root weight; LR, length of root; NFR, numbers of fibrous roots; RFR, ratio of root surface covered with fibrous roots; PCR, P. brassicae content in roots.*

*^∗∗^P = 0.01.*

### SNP Detection and Genetic Map Construction

**FIGURE 2:**
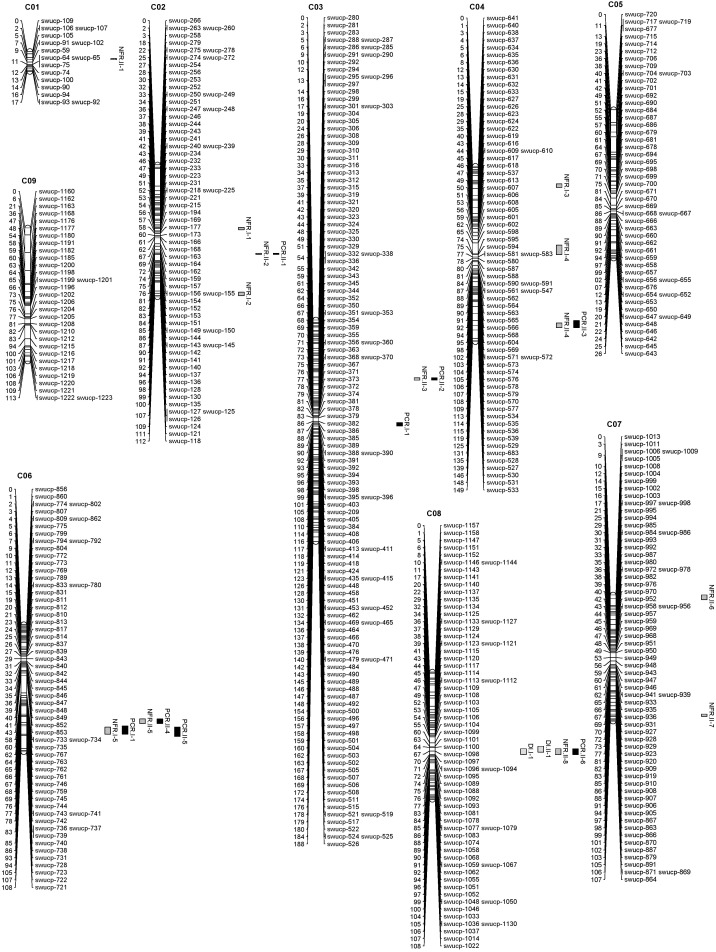
The *Brassica oleracea* genetic linkage map and quantitative trait loci (QTLs) for disease incidence (DIC), the number of fibrous roots (NFRs), and the *P. brassicae* content in roots (PCR).

Among 52,157 SNPs in the *Brassica* 60K SNP array, 21,646 (41.5%) could be aligned to the *B. oleracea* genome. These SNPs distributed unevenly on the nine chromosomes of *B. oleracea*, with the highest number on chromosome C03 (3,558) and the fewest on chromosome C05 (1,264), being in accordant with the chromosome length of *B. oleracea*. Among the C-genome SNPs, 3,218 (14.9%) were polymorphic between the two parents, forming 615 bins. After removing the serious missing bins, the skewed bins (*P*≤ 0.01) and the redundant bins, 565 bins were allocated into nine linkage groups (LGs), with the fewest on C01 (16 bins) and the most number on C03 (130 bins) (**Table 3**). The lengths of LGs ranged from 17.2 to 187.7 cM, summed in a total length of 1,027.98 cM, with an average distance of 1.82 cM between neighboring bins (**Figure 2** and **Table 3**). The bins showed general coincidence between their genetic and physical positions for all LGs except C01 which has a limited number of bins.

**Table 3:** Overview of bins on the nine linkage groups of *Brassica oleracea.*

Linkage group	Length (cM)	No. of bins	Average interval (cM)	No. of skewed bins (a/b)^∗^	Percentage of segregation distortion (%)
C01	17.16	16	1.07	8 (2/6)	50
C02	112.23	69	1.63	2 (1/1)	2.9
C03	187.69	130	1.44	9 (7/2)	6.92
C04	149.14	69	2.16	2 (2/0)	2.9
C05	125.69	53	2.37	6 (3/3)	11.32
C06	108.08	62	1.74	3 (3/0)	4.84
C07	106.95	68	1.57	18 (13/5)	26.47
C08	108.35	67	1.62	1 (1/0)	1.49
C09	112.69	31	3.64	3 (1/2)	9.68
Total	1027.98	565	–	52 (33/19)	–
Average	114.22	62.78	1.82	5.78 (3.67/2.11)	9.2

*^∗^a and b represent the numbers of bins skewed toward the female parent ‘GZ87’ and male parent ‘263,’ respectively.*

### QTLs for CR-Associated Traits

**FIGURE 3:**
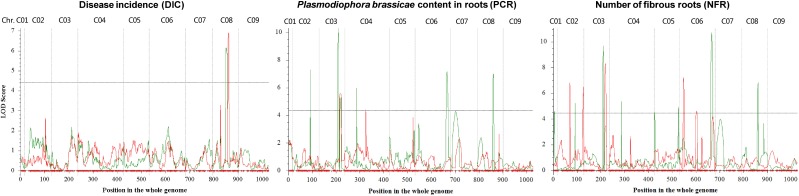
Quantitative trait loci scanning for clubroot resistance associated traits in *B. oleracea* in the first (red) and the second replication (green).

With significant LOD thresholds of 4.4, 4.5, and 4.4 which were set the by 1,000-permutation test in IciMapping, 2, 13, and 8 QTLs for DIC, NFR, and PCR, were detected respectively (**Figures 2**, **3** and **Table 4**), individually explaining 6.1–17.7% of the phenotypic variation. Overlaps were detected between two rounds, such as *DIC.I-1* and *DIC.II-1*, and *PCR.I-2* and *PCR.II-5*. Overlaps were further detected among traits, including one three-traits-overlapped QTL on LG C08 and four overlapped QTLs between NFR and PCR on LG C03, C04, and C06. The QTLs on LG C08 (*DIC.I-1*, *DIC.II-1*, *NFR.II-8*, and *PCR.II-6*) exhibited 10.2–17.8% of the total phenotypic variation, with an overlapped genetic range of 64.5–67.5 cM which corresponding to a 0.6 Mb interval (32.7–33.3 Mb) on chromosome C08. It is worth mentioning that two overlapped QTL regions on LG C06 between NFR and PCR also exhibited high *R*^2^ values. In details, *NFR.I-5*, *PCR.I-2*, and *PCR.II-5* overlapped on 86.5–92.5 cM which corresponding to 1.98–3.54 Mb on chromosome C06, and *NFR.II-5* and *PCR.II-4* overlapped on 77.5–83.5 cM, being corresponded to 3.26–3.94 Mb of C06.

**Table 4:** Quantitative trait loci (QTLs) for the number of disease incidence (DI), fibrous roots (NFR), the root surface covered with fibrous roots (RFR), and the *Plasmodiophora brassicae* content in roots (PCR) in *Brassica oleracea.*

QTL	Chromosome	Peak pos. (cM)	Confidence interval (cM)	Left marker	Right marker	LOD score	Genetic effects^∗^			Phenotypic variation explained (%)
							*a_F_*	*a_M_*	*d*	
DIC.I-1	8	67	64.5–69.5	swucp-1098	swucp-1097	6.91	0.03	0.14	0.02	17.66
NFR.I-1	2	53	52.5–54.5	swucp-225	swucp-221	6.79	-0.05	0.01	-0.31	10.46
NFR.I-2	2	109	107.5–110.5	swucp-124	swucp-121	6.49	-0.06	0.04	-0.29	9.8
NFR.I-3	4	15	14.5–17.5	swucp-632	swucp-633	7.22	0.04	-0.03	0.32	11.29
NFR.I-4	4	71	66.5–74.5	swucp-598	swucp-595	4.62	0.01	0.01	-0.27	7.92
NFR.I-5	6	91	86.5–92.5	swucp-738	swucp-731	8.3	0.21	-0.28	0.05	14.71
PCR.I-1	3	88	86.5–89.5	swucp-386	swucp-385	4.4	-3.69	3.69	10.54	12.98
PCR.I-2	6	89	85.5–92.5	swucp-738	swucp-731	5.58	-3.97	13.26	-0.09	17.76
DIC.II-1	8	64	62.5–67.5	swucp-1101	swucp-1098	7.28	0.00	0.16	0.02	14.8
NFR.II-1	1	6	5.5–6.5	swucp-105	swucp-91	4.6	0.06	-0.19	0.11	6.1
NFR.II-2	2	75	74.5–75.5	swucp-157	swucp-156	5.21	0.04	-0.01	-0.3	8.06
NFR.II-3	3	50	48.5–50.5	swucp-330	swucp-329	5.35	-0.01	-0.29	-0.04	7.58
NFR.II-4	4	134	130.5–136.5	swucp-529	swucp-528	10.71	0.13	-0.01	0.41	16.74
NFR.II-5	6	83	79.5–83.5	swucp-742	swucp-736	9.68	0.01	-0.41	0.02	15.49
NFR.II-6	7	0	0.0–3.5	swucp-1013	swucp-1011	4.53	-0.03	-0.09	-0.24	6.13
NFR.II-7	7	102	100.5–102.5	swucp-887	swucp-879	4.94	0.25	0.08	0.06	6.7
NFR.II-8	8	69	64.5–69.5	swucp-1098	swucp-1097	6.83	0.05	-0.32	0.05	10.17
PCR.II-1	2	75	74.5–75.5	swucp-157	swucp-156	7.29	-2.9	0.43	13.33	12.02
PCR.II-2	3	50	48.5–50.5	swucp-330	swucp-329	5.96	0.05	11.39	2.07	9.04
PCR.II-3	4	134	130.5–136.5	swucp-529	swucp-528	7.15	-3.83	1.39	-12.03	11.07
PCR.II-4	6	83	79.5–83.5	swucp-742	swucp-736	10.31	-0.5	15.26	-2.57	17.49
PCR.II-5	6	93	86.5–94.5	swucp-738	swucp-731	5.28	-10.86	0.74	-0.14	7.97
PCR.II-6	8	67	64.5–69.5	swucp-1098	swucp-1097	7.01	0	12.64	0.8	11.11

*^∗^a_F_ and a_M_ represent the additive genetic effects of ‘GZ87’ and ‘263,’ respectively; d represents dominant effects.*

### Comparative Analysis Among CR Loci

According to Bolbase^2^ which provides information on syntenic regions between *B. oleracea* and *B. rapa* (Yu et al., 2013), a QTL interval on C03 (6.28–6.32 Mb, overlapped by *NFR.II-3* and *PCR.II-2*) in the present study was partially syntenic to 0.59–6.22 Mb on A02 of *B. rapa* where located the *CRc*- and *Pb(Anju)2*- linked marker ‘m6R’ (from 2,112,653 to 2,113,153 bp) (Sakamoto et al., 2008; Nagaoka et al., 2010; Lee et al., 2016). A QTL on C07 (*NFR.II-7*) was found to be syntenic to 11,077,112–11,414,470 bp on A08 of *B. rapa*, being partially overlapped with a reported CR loci *Crr1* which was fine mapped to 10,692,602–11,617,700 bp on A08 in *B. rapa* (Hasan and Rahman, 2016). The three important regions on C08 and C06 identified no accordance with reported CR loci.

## Discussion

In the past, QTLs were mainly identified based on genetic linkage map constructed by DNA molecular markers such as RAPD, RFLP, AFLP, and SSR markers (Landry et al., 1992; Voorrips et al., 1997; Piao et al., 2002, 2004; Suwabe et al., 2006), however, this approach is high-input but low-output in identifying candidate genes. Comparatively, the *Brassica* SNP microarray which has been widely applied in *B. napus* could provide high-density genetic maps and thus greatly narrowed the QTL regions for interested traits (Liu et al., 2013; Wei et al., 2016). Since no *B. oleracea*-specific SNP microarray was available before this study, the 60K *Brassica* SNP microarray which consisted of probes from both A and C genomes (Clarke et al., 2016) was used in the present study. Although nearly 60% (30,511) SNPs were filtered out (could not be aligned to C genome), the 3,218 polymorphic sites from the remaining 40% (21,646) SNPs still enabled us to construct a high-density genetic map of *B. oleracea* which spanned 1028 cM of the *B. oleracea* genome with an average of 1.82 cM (0.67 Mb) between neighboring bins. Given the high-throughput and time-saving nature of SNP array, this approach will dramatically accelerate the process of QTL fine-mapping in *B. oleracea*.

In the studies associated with CR in *Brassica*, disease index was the most widely used indicator for CR level (Crisp et al., 1989; Landry et al., 1992; Matsumoto et al., 1998; Carlsson et al., 2004; Hirai et al., 2004; Hirai, 2006; Dixon and Robinson, 2010; Hatakeyama et al., 2013; Kato et al., 2013; Liu et al., 2013; Hasan and Rahman, 2016; Lee et al., 2016; Huang et al., 2017; Yu et al., 2017). However, it was hard to judge the disease index of vegetative propagates in the present study since the disease grade of each copy was difficult to class due to the lack of main roots in vegetative plants and the difficulties on distinguishing clubroot tubers from exogenous-hormone-induced calluses in tissue culture. Nevertheless, it was relatively easier to discriminate diseased plants from healthy ones, thus disease incidence was recorded, though its accuracy to indicate the resistance level of host might be not as high as that by using disease index. As supplements of disease incidence, we measured several traits which might be potentially correlated with the resistance of vegetative plants, and we found the *P. brassicae* content in roots was highly correlated with DIC, being consistent with that in *B. rapa* (*r* = 0.95) (Zhu et al., 2015). In addition, the NFR was found to be moderately correlated with DIC and high correlated with PCR. Therefore, the two CR-associated traits particular PCR could be used to indicate the resistance level of plants when needed, for example, in the plants without main roots.

Clubroot-resistant genes or loci were widely identified in *B. rapa* (Hirai et al., 2004; Piao et al., 2004; Suwabe et al., 2006; Sakamoto et al., 2008; Huang et al., 2017; Yu et al., 2017) and *B. oleracea* (Landry et al., 1992; Voorrips et al., 1997; Rocherieux et al., 2004; Moriguchi et al., 2010; Nagaoka et al., 2010; Lee et al., 2016). It was hypothesized that the CR of *B. rapa* was possibly controlled by qualitative plus quantitative loci (Piao et al., 2002; Suwabe et al., 2006; Sakamoto et al., 2008), while CR trait in *B. oleracea* might be controlled by QTLs in a quantitative manner (Crisp et al., 1989; Voorrips et al., 1997; Piao et al., 2009; Tomita et al., 2013). In the present study, although only one genetic region on C08 was identified for DIC, many QTLs were identified for the other two CR-associated traits. These QTLs including the two QTLs for DIC explained limited phenotypic variations (6.1–17.8%), suggesting the quantitative nature of these traits and indicated that the pyramiding of these loci may confer durable CR in *B. oleracea*.

Two possible overlaps of our QTLs were detected with previous reported loci after deep comparison of our regions with other studies which provided direct or indirect (primer sequences of linked markers were then blasted to reference genome to get the physical positions) genomic information of CR loci (Matsumoto et al., 1998; Saito et al., 2006; Suwabe et al., 2006; Sakamoto et al., 2008; Nagaoka et al., 2010; Hatakeyama et al., 2013; Kato et al., 2013; Hasan and Rahman, 2016; Lee et al., 2016), however, the QTLs in these two regions only explained 6.7–10.3% of the total phenotypic variation. It is interesting that no overlap was detected for reported CR loci with the three important regions found from C08 and C06 in our study. Although A08 of *B. rapa* was reported to carry important CR QTLs such as *Crr1* and *Rcr9* (Hasan and Rahman, 2016; Yu et al., 2017), they are not syntenic to our region on C08. These data suggest that the CR in our resistant *B. oleracea* GZ87 is possibly controlled by some novel loci. In each of the three genetic regions, we found several pathogen-responsive genes, including genes encoding receptor like proteins (R proteins), receptor binding proteins, auxin efflux transporters, auxin-responsive proteins, RHD3s (root hair defective 3, required for cell expansion and normal root hair development), pathogen-responsive factors (alpha-dioxygenase, calcium-binding, thaumatins, et al.) and transcriptional factors (MYB, ERF, C2H2, HD-ZIP, et al.). Further study will be carried out to identify the candidate genes and develop linked markers for a pyramiding purpose, since accumulation of QTLs could confer broad-spectrum CR in *B. oleracea* (Tomita et al., 2013).

## Author Contributions

LP, LZ, QL, and DW conducted the lab experiments and data analysis. XR and HS conducted the field experiments. LP, JM, JS, and WQ wrote the manuscript. JM, JS, and WQ directed the project.

## Conflict of Interest Statement

The authors declare that the research was conducted in the absence of any commercial or financial relationships that could be construed as a potential conflict of interest.

## Footnotes

1 ftp://brassicadb.org/Genomes/Brassica_oleracea/V1.1/

2 http://www.ocri-genomics.org/bolbase/
